# Physical Diagnosis

**Published:** 1892-10

**Authors:** 


PHYSICAL DIAGNOSIS—A Guide to Methods of Clinical Investigation. By G. A. Gibson, M.D., D. Sc., F. R. C. P. Ed. Lecturer on the Principles and Practice of Medicine in the Edinburgh Medical School; and William Russell, M.D., F. R. C. P. Ed., Pathologist to the Royal Infirmary of Edinburgh. With one hundred and one illustrations. Pages, 379. New York: D. Appleton & Co., 1891.

The authors of the above work have succeeded in selecting the most practical points in physical diagnosis, giving the methods of clinical investigation in a clear and succinct manner. Being practical teachers themselves in the great schools of Edinburgh and Glasgow, they have given the results of years of experience in this every-day branch of medicine. The JOURNAL is pleased to note that the fine abilities of Dr. Wm. Keiller, Professor of Anatomy in the Medical Department of the University of Texas, has been called into requisition in furnishing the diagrams illustrating topographical anatomy, etc., in this splendid work. B.

